# Ghrelin Receptors in Non-Mammalian Vertebrates

**DOI:** 10.3389/fendo.2013.00081

**Published:** 2013-07-17

**Authors:** Hiroyuki Kaiya, Kenji Kangawa, Mikiya Miyazato

**Affiliations:** ^1^Department of Biochemistry, National Cerebral and Cardiovascular Center Research Institute, Osaka, Japan; ^2^National Cerebral and Cardiovascular Center Research Institute, Osaka, Japan

**Keywords:** ghrelin, ghrelin receptor, GHS-R, GHS-R-like receptor, fishes, amphibians, reptiles, birds

## Abstract

The growth hormone secretagogue-receptor (GHS-R) was discovered in humans and pigs in 1996. The endogenous ligand, ghrelin, was discovered 3 years later, in 1999, and our understanding of the physiological significance of the ghrelin system in vertebrates has grown steadily since then. Although the ghrelin system in non-mammalian vertebrates is a subject of great interest, protein sequence data for the receptor in non-mammalian vertebrates has been limited until recently, and related biological information has not been well organized. In this review, we summarize current information related to the ghrelin receptor in non-mammalian vertebrates.

## General Introduction

As implied by their name, growth hormone secretagogues (GHSs), which are artificial derivatives of enkephalin, exhibit growth hormone (GH)-releasing activity (1). Some of these GHSs also stimulate appetite in mammals (2). In 1996, Howard et al. (3) discovered a G-protein-coupled receptor (GPCR) with seven transmembrane domains (TMDs) in humans and pigs, and found that GHSs bound to this receptor and elicited an increase in the intracellular Ca^2+^ concentration of cells in which it was stably expressed. They named this receptor the GHS-receptor type-1a (GHS-R1a); in addition, they found an alternative splice variant of the receptor that lacked the Ca^2+^ signaling capacity and named it GHS-R type-1b (GHS-R1b). The mammalian GHS-R gene (*ghsr*) comprises two exons separated by one intron (4, 5). GHS-R1a comprises 366 amino acids (AAs), where the first exon (exon 1) encodes the first 265 AAs from TMD 1–5, and the second exon (exon 2) encodes the remaining 101 AAs from TMD 6 and 7. In contrast, the alternative splice variant of *ghsr*, GHS-R1b, is formed from the first exon and part of the intron. Thus, the protein sequence of the entire 289-AA GHS-R1b is identical to GHS-R1a from the N-terminal end to TMD 5.

Extensive investigations were performed to identify the endogenous ligand for the orphan GHS-R1a following discovery of the receptor, and reverse pharmacology facilitated the identification of a natural ligand in 1999 by Kojima et al. (6). The peptide ligand, which contains 28 AAs, was isolated from stomach extracts of rats and named “ghrelin.” Ghrelin has a unique fatty acid modification on its N-terminal third serine (Ser3), with an *n*-octanoyl group linked to the hydroxyl group of Ser3. This modification is essential for the binding of ghrelin to the receptor (7) and for eliciting various physiological actions. After the discovery of its endogenous ligand, GHS-R1a was found to mediate various physiological functions of ghrelin: neuroendocrine function; appetite regulation; cardiovascular function; gastro-entero-pancreatic function; glucose metabolism; and cell functions including apoptosis, proliferation, and differentiation (8–10).

In non-mammalian vertebrates, GHSs affect the regulation of GH release and of appetite in fish and birds (11–14), suggesting the presence of an endogenous ghrelin-like substance and a corresponding receptor system. We first isolated ghrelin from a non-mammalian vertebrate, the bullfrog (15). Subsequently, ghrelin was determined to be present in various non-mammalian vertebrates, and its physiological effects were gradually revealed [for reviews, see Ref. (16, 17)]. However, investigations of non-mammalian ghrelin receptors still lag behind those on mammalian ghrelin receptors. In this review, we summarize our recent work and those of others on ghrelin receptors in non-mammalian vertebrates and provide a comprehensive discussion of their general features.

## Classification and Nomenclature of Ghrelin Receptors

**Figure 1:**
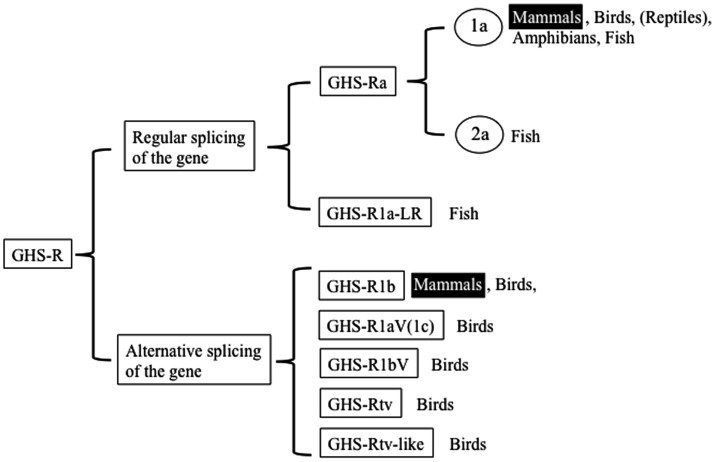
**Classification of ghrelin receptors**. Receptors that exist in mammals are highlighted.

We begin by describing the nomenclature for the ghrelin receptors in mammals, because the nomenclature for the receptors in non-mammalian vertebrates is more complicated and various names have been used based on the presence of splice variants, paralogs, and different AA lengths. In the first description provided by Howard et al. (3), GHS-R1a was defined as a functional receptor induced by agonist-dependent intracellular Ca^2+^, and GHS-R1b as a splice variant of unknown function. They classified them simply as “a” and “b” because their sequences and functions differed. Thus the names are based on the sequence and structure: “GHS-R1” refers to the receptor with a “type-1” AA sequence, “a” signifies “activated by ghrelin or GHSs,” and “b” indicates “a splice variant of *ghsr*” which contains the first exon and an unspliced intron that continues the coding sequence in the mRNA and terminates at a stop codon within the intron. The International Union of Pharmacology Committee on Receptor Nomenclature and Drug Classification has accepted “GHS-R1a” as the name for the functional ghrelin receptor (18). Hence, two GHS-Rs exist in mammals: GHS-R1a, which is derived from regular splicing of the gene; and GHS-R1b, which originates from alternative splicing of the gene (Figure 1). On the basis of these names, we describe the naming of the receptors in non-mammalian vertebrates as follows.

**Figure 2:**
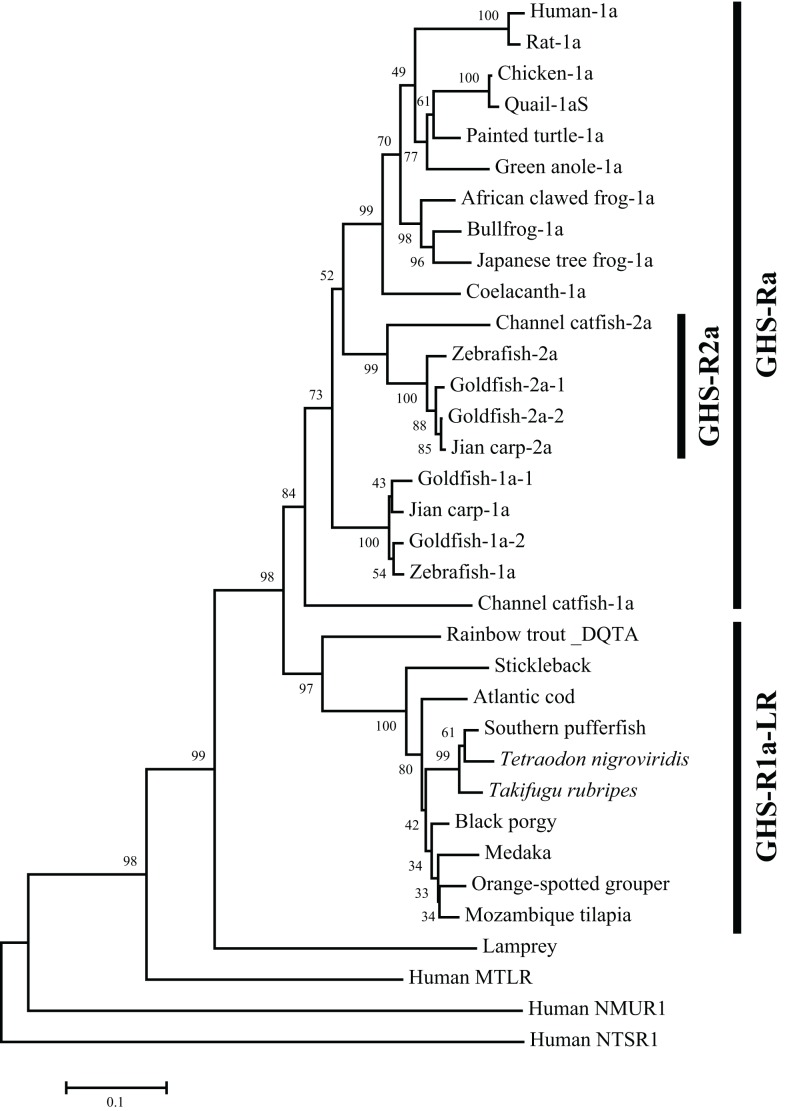
**Phylogenetic tree of GHS-Ra and GHS-R1a-LR in non-mammalian vertebrates**. The phylogenetic tree was constructed by using the neighbor-joining method with MEGA4 (http://www.megasoftware.net/). The numbers on the branch points are the bootstrap values (as percentages based on 2000 replicates). The scale bar indicates the average number of substitutions per position (a relative measure of evolutionary distance). Receptors for human motilin (MTLR), neuromedin-U (NMUR1), and neurotensin (NTSR1) were used as the outgroup.

The non-mammalian GHS-Rs are also roughly divided into two types: (i) an isoform that arises from regular splicing of the gene and (ii) an isoform derived from alternative splicing of the gene (Figure 1). The former is further classified into two isoforms (Figure 1): one denotes an isoform that we designated “GHS-Ra,” which has structural properties similar to those of the mammalian GHS-R1a and is activated by ghrelin and GHSs. GHS-Ra is further divided into two paralogs “1a” and “2a,” where “GHS-R2a” refers to the receptor with a “type-2” AA sequence distinct from that of GHS-R1a and whose existence is confirmed only in specific fish. The other denotes another isoform that we designated “GHS-R1a-like receptor (GHS-R1a-LR),” which has structural features that differ from those of GHS-Ra and for which intracellular Ca^2+^ increase in response to ghrelin or GHS treatment is either small or not confirmed. This distinction between GHS-Ra and GHS-R1a-LR is evident in the phylogenetic analysis based on the AA sequences of ghrelin receptors (Figure 2).

The isoforms derived from alternative splicing of the gene are divided into five types: 1b, 1aV (1c), 1bV, tv, and tv-like receptors. These receptors are formed by different modes of alternative splicing and have distinct structures.

## Non-Mammalian Vertebrate Species with Sequenced Ghrelin Receptors

**Figure 3:**
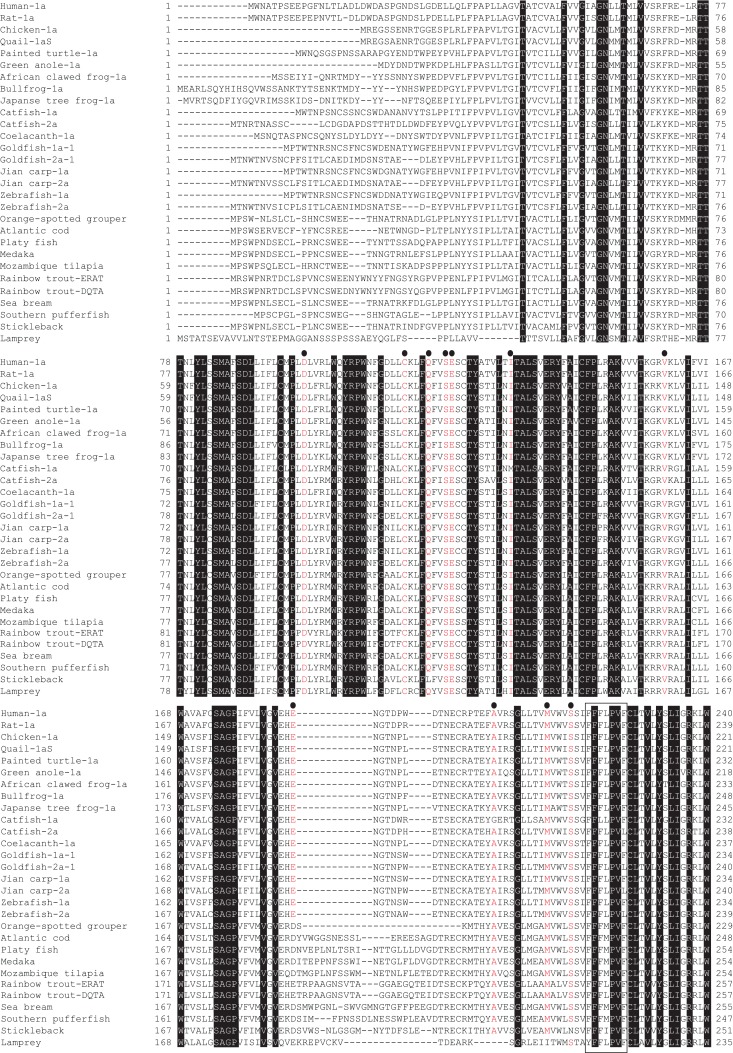

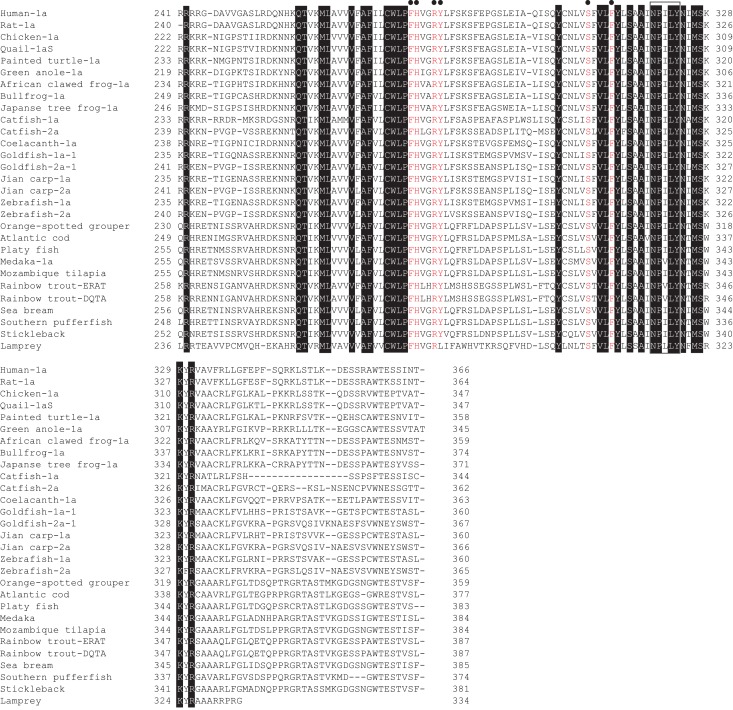
**GHS-R1a, 2a and GHS-R1a-LR proteins in non-mammalian vertebrates**. Dense shading indicates common amino acids (AAs) in all species. Red letters under black circles represent specific AAs related to ligand binding, selectivity, and constitutive activity of GHS-R1a and 2a. Boxes show the typical motifs of the G-protein-coupled receptor transmembrane domains 5 and 7. Sequences were aligned using GENETYX-Mac version 15.0.1.

We have summarized the non-mammalian vertebrates for which the cDNA or genes of GHS-R have been identified and made available in public databases in Table 1 (fish) and Table 2 (reptiles, amphibians, and birds). The AA sequences of GHS-R1a, 2a; GHS-R1a-LR; and their multiple alignments are shown in Figure 3.

**Table 1:** **Ghrelin receptor and ghrelin receptor-like receptor in fish**.

Species	Name	type	Accession number	cDNA length (bp)	Number of amino acids	Reference	Remarks
**FISH**							
*Salmo salar*	Atlantic salmon	1a	GQ373171	669	220	(65)	Partial sequence
		1b		303	100	(65)	Partial sequence
*Acanthopagrus schlegelii*	Black porgy	1a	AY151040	1723	385	(28)	
		1b	AY151041	1821	295	(28)	
		GHS-R1 gene	AY151041	1821	-	(28)	
		5′-Flanking region	AY509196	2157	-	(68)	
*Ictalurus punctatus*	Channel catfish	1a	FJ707319	1632	344	(39)	
		2a	FJ707321	1490	362	(39)	
		1b	FJ707320	1877	307	(39)	
*Carassius auratus*	Goldfish	1a-1	AB504275	1083	360	(22)	
		1a-2	AB504276	1083	360	(22)	
		2a-1	AB504277	1104	366	(22)	
		2a-2	AB504278	1101	367	(22)	
		GHS-R1-1 gene	AB555555	1722	-	(22)	
		GHS-R1-2 gene	AB555556	2059	-	(22)	
		GHS-R2-1 gene	AB555557	2012	-	(22)	
		GHS-R2-2 gene	AB555558	2859	-	(22)	
*Oreochromis mossambicus*	Mozambique tilapia	1a-LR	AB361053	1584	384	(23)	
		1a-LR	EU334002	1627	384	(96) (direct submission)	
		GHS-R1-LR gene	AB361055	1815	-	(23)	
		GHS-R1-LR gene	EU910220	3253	-	(97) (direct submission)	
		1b	AB361054, EU334003	897; 1858	298	(23, 96) (direct submission)	
*Oreochromis niloticus*	Nile tilapia	1a-LR	ENSONIT00000001069	1158	384	Ensembl Genome Browser	
*Oreochromis urolepis*	Wami tilapia	1a-LR	EU243664	1646	384	(96) (direct submission)	
		Promotor region	FJ217700	1619		(97) (direct submission)	
		1b	EU243665	1877	298	(96) (direct submission)	
*Epinephelus coioides*	Orange-spotted grouper	1a-LR	Not deposited	1512	383	(45)	
		1b	Not deposited	1703	303	(45)	
*Spheroides nephelus*	Pufferfish	78B8 gene 1b?	AF082209 AF082209	1455	374; 313	(27)	
*Tetraodon nigroviridis*	Pufferfish	1a-LR gene	ENSTNIG00000006665		394	Ensembl Genome Browser	
*Takifugu rubripes*	Japanese pufferfish	1a-LR			377	Fugu genome project (http://genome.jgi-psf.org/)	
*Oncorhynchus mykiss*	Rainbow trout	1a-LR (DQTA/LN-rype)	AB362479	1673	387	(23)	
		1a-LR (ERAT/IS-type)	AB362480	1164	387	(23)	
		1b (DQTA/LN-rype)	AB362481	2234	297	(23)	
		1b (DQTA/LN-rype), gene	AB479381		300	(23)	
		1b (ERAT/IS-type), gene	AB362482	1688	297	(23)	
*Danio rerio*	Zebrafish	1a	NM_001146272	1803	360	(93)	
		2a	XM_002666671	1098	365	(16)	
*Gadus morhua*	Atlantic cod	1a-LR	ENSGNOT00000014265		377	Ensembl Genome Browser	
*Latimeria chalumnae*	Coelacanth	1a-LR	ENSLACT00000015868		363	Ensembl Genome Browser	
*Petromyzon marinus*	Sea lamprey	1a-LR	ENSMAT00000007290		344	Ensembl Genome Browser	
*Oryzias latipes*	Japanese medaka	1a-LR	ENSORLT00000014679		384	Ensembl Genome Browser	
*Xiphophorus maculatus*	Southern platyfish	1a-LR	ENSFM005 00000270343		383	Ensembl Genome Browser	
*Gasterosteus aculeatus*	Three-spined stickleback	1a-LR	ENSGAT00000014515		381	Ensembl Genome Browser	
*Cyprinus carpio jian*	Jian carp	1a	HM191491	1083	360	(99) (direct submission)	
		1b	HM191493	1083	360	(99) (direct submission)	
		1a′	HM191492	892	184	(99) (direct submission)	
		1b′	HM191494	892	184	(99) (direct submission)	
		GHS-R1a gene	HM191495	2789		(99) (direct submission)	
		GHS-R2a gene	HQ162474	2064		(99) (direct submission)	
		GHS-R2b gene	HQ162475	2129		(99) (direct submission)	
*Cyprinus carpio*	Common carp	1b	JN392468	1968	360	(100) (direct submission)	

*NCBI (http://www.ncbi.nlm.nih.gov/); NCBI genome database (http://www.ncbi.nlm.nih.gov/genome/); Ensembl Genome Browser (http://www.ensembl.org/index.html)*.

**Table 2:** **Ghrelin receptor and ghrelin receptor-like receptor in reptiles, amphibians, and aves**.

Species	Name	type	Accession number	cDNA length (bp)	Number of amino acids	Reference	Remarks
**REPTILES**							
*Anolis carolinensis*	Green anole	1a	XM_003218148	1038	345		
*Chrysemys picta bellii*	Western painted turtle	1a	JH584696		358	Ensembl Genome Browser	
124 Species of *Squamata*		1a	JN880998–JN881119				Partial sequence
**AMPHIBIAN**							
*Rana caiesbeiana*	Bullfrog	1a	AB626731	1125	374	(19)	
*Hylajaponica*	Japanese tree frog	1a	AB626732	1116	371	(19)	
*Xenopus tropicalis*	Tropical clawed toad	1a	XM_002931572	1080	359	NCBI genome database	
**AVES**							
*Gallus gallus*	Chicken	1a	NM_204394	1699	347	(29, 30)	
		GHS-R1 gene	AB095994	4121	–	(30)	
		1aV	AB095996	1703	331	(30)	
		1b	AB095997	1351	276	(30)	
		1c	AJ309543	646	215	(29)	Partial sequence
		tv	Not deposited			(31, 33)	
*Anas platyrhynchos*	Mallard	1a gene	FJ194548	3717	245	(44)	Partial sequence
*Coturnix japonica*	Japanese quail	1a-L	AB469019	1308	354	(32)	
		1a-S	AB469019	1287	347	(32)	
		1aV-L	AB469020	1260	338	(32)	
		1b-L	AB469022	909	302	(32)	
		1bV-L	AB469021	930	309	(32)	
		tv-like	AB490327	2661	251	(32)	Partial sequence
*Meleagris gallopavo*	Turkey	1a	NW_003435736	3965	473	NCBI genome database	Partial sequence
*Taeniopygia guttata*	Zebra finch	1a	XM_002193702	864	287	NCBI genome database	Partial sequence

*NCBI (http://www.ncbi.nlm.nih.gov/); NCBI genome database (http://www.ncbi.nlm.nih.gov/genome/); Ensembl Genome Browser (http://wwwensembl.org/index.html)*.

Many GHS-Rs have been identified in non-mammalian vertebrates, and the most of the GHS-R types that have been found are present in fish (19 species). With the recent identification of a GHS-R in bullfrog and Japanese tree frog (19), we now know the GHS-Rs for three kinds of frogs, including African clawed frogs. In reptiles, there are no reports about GHS-Rs at present, although the Ensembl genome database search (http://www.ensembl.org/index.html) yields the GHS-R1a gene for the green anole (*Anolis carolinensis*) and painted turtle (*Chrysemys picta bellii*). Very recently, massive numbers of partial nucleotide sequences (approximately 450-bp encoding a 150-AA protein) of GHS-R have been registered for 124 species of *Squamata*, including snakes and Iguanidae, by Wiens et al. (98) at Stony Brook University in the NCBI database. In birds, GHS-Rs have been found in five species.

## Structural Features of the Ghrelin Receptor in Non-Mammalian Vertebrates

Three features are prominent in non-mammalian GHS-Rs: (1) the presence of paralogs in a few species of teleosts; (2) two isoforms, GHS-Ra and GHS-R1a-LR; and (3) avian-specific alternative splice forms of GHS-R (Figure 1). Further details are provided below (see also Classification and Nomenclature of Ghrelin Receptors).

### Presence of paralogs in only a few species of teleosts

The GHS-Ra paralog GHS-R2a is found only in a limited number of teleosts, and little is known about the presence of GHS-R paralogs in other vertebrates. GHS-R2a has an AA sequence that is approximately 70% identical to that of GHS-R1a. At present, this receptor has been identified in Cypriniformes such as goldfish, zebrafish, and carp, and in channel catfish in the order Siluriformes (Figures 2 and 3). The two isoforms are encoded by different genes (i.e., the zebrafish GHS-R1a and 2a genes are located separately on chromosomes 4 and 24, respectively), which are considered to have diverged via the third round of whole-genome duplication (3R-WGD) that occurred in the ray-finned fish lineage (20, 21).

In addition, isoforms with approximately 95% identity have been found in goldfish (Cypriniformes) and rainbow trout (Salmoniformes). In goldfish, there are two paralogs each for GHS-R1a and 2a: GHS-R1a-1, 1a-2, 2a-1, and 2a-2 (Figures 2, 3, and 5). Each receptor originated from a separate gene demonstrated to have a different intron sequence (22). In the rainbow trout, two paralogous sequences, namely the DQTA/LN-type and ERAT/IS-type, have been identified (23) (Figure 3). Their names indicate AA substitutions at D20E, Q32R, T54A, A62T, L168I, and N264S. These two receptor sequences are known to be derived from at least three distinct genes (the DQTA/LN-type derives from two genes and the ERAT/IS-type originates from one gene), on the basis of analyses of an intron sequence of each receptor (23). These paralogs of goldfish and rainbow trout are considered to have originated from polyploidization events that occurred after 3R-WGD (24) and tandem duplication of the genes, which also affected the opsin gene in these species (25). The presence of multiple paralogs may be a peculiar characteristic of *Ostariophysi* and *Protacanthopterygii* in euteleosts (20, 21).

### Two ghrelin receptor isoforms: GHS-Ra and GHS-R1a-LR

**Figure 4:**
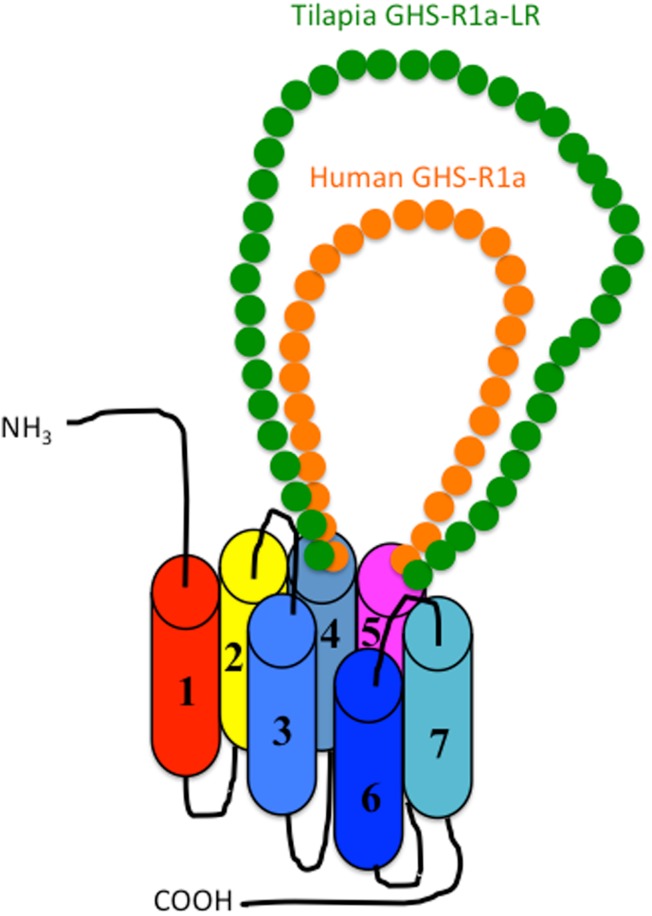
**Schematic diagram of the second extracellular loop (ECL2) in the GHS-R1a and GHS-R1a-like receptors**. Human GHS-R1a, which has a short ECL2 (orange), and tilapia GHS-R1a-like receptor (GHS-R1a-LR), which has a long ECL2 (green), are shown as representative examples. The length of ECL2 in human GHS-R1a is 28 amino acids (AAs), whereas the ECL2 in tilapia GHS-R1a-LR is 43 AAs. Each receptor is classified in a different branch of the phylogenetic tree (Figure 2). GHS-Ra, which includes GHS-R1a or 2a, is found in tetrapods including chickens (birds), mammals, reptiles, and amphibians, as well as some bony fishes such as Coelacanthiformes, Cypriniformes (e.g., goldfish, carp, and zebrafish), and Siluriformes (channel catfish). These animal species have the receptor with the short ECL2. In contrast, GHS-R1a-LR is found only in a fish group that includes Perciformes such as tilapia, Gasterosteiformes such as stickleback and medaka, Tetraodontiformes such as pufferfish, and Salmoniformes such as rainbow trout.

As shown in Figure 1, there are two isoforms in non-mammalian vertebrates: GHS-Ra and GHS-R1a-LR. GHS-Ra includes GHS-R1a and 2a. Tetrapods including mammals, birds, reptiles, and amphibians have GHS-R1a, whereas some bony fish such as Coelacanthiformes, Cypriniformes (e.g., goldfish, carp, and zebrafish), and Siluriformes (e.g., channel catfish) have both GHS-R1a and 2a. GHS-R1a-LRs show considerable AA identity to GHS-R1a, but have a unique structural feature not found in any tetrapod: the second extracellular loop (ECL2) that connects TMD 4 and 5 is notably longer than that of GHS-R1a (Figure 4). In addition, GHS-R1a-LRs have the characteristic that ghrelin or GHS treatment either does not increase intracellular Ca^2+^ (23, 26) or requires pharmacological doses to activate the receptor (27, 28). This type of receptor is seen in a limited number of fish classified as Percomorpha within the superorder *Acanthopterygii*, which is the most evolutionally advanced group of teleosts, including Perciformes such as black porgy and tilapia, Gasterosteiformes such as stickleback and medaka, Tetraodontiformes such as pufferfish, and Salmoniformes such as rainbow trout (Figure 3). An exception is the orange-spotted grouper, which belongs to Perciformes but has an ECL2 that is not long (Figure 3). These species have some morphological characteristics such as a highly mobilized upper jaw, a respiratory tract not linked to the swim bladder, and a splinter article in their fins. Salmoniformes belong to *Protacanthopterygii*, which contains a number of moderately advanced teleosts. This evolutionary background may be reflected in the molecular evolution and structure of the ghrelin receptor.

A partial sequence similar to that of the ghrelin receptor was found in a database for the sea lamprey (*Petromyzon marinus*). This receptor could not be placed at the branch of GHS-Ra or GHS-R1a-LR in the phylogenetic analysis (Figure 2). The sea lamprey belongs to the group Cyclostomata in the class Agnatha, which is a class of fish with the characteristics of ancient basal vertebrates. Therefore, the receptor in the sea lamprey may contain ancestral characteristics of the ghrelin receptor.

### Avian-specific GHS-Rs

Birds have specific alternative spliced forms of GHS-R other than GHS-R1b, i.e., 1aV (or 1c), 1bV, tv, and tv-like receptor (29–32), which are generated by differential modes of splicing from GHS-R1b. GHS-R1aV (30) and GHS-R1c (29) are identical receptors found in chickens. Here, “V” is considered to mean “variant” (30), whereas Geelissen et al. (29) used the designation “c” to indicate an isoform different from “a” or “b.” We proposed that GHS-R1c should be referred to as GHS-R1aV because the receptor is identical to GHS-R1a with the exception that it lacks 16 AAs (46 bp) in TMD 6 (16). GHS-R1bV is found in quail. Its C-terminal part differs from that of GHS-R1b, and an AA sequence that differs from 1b is translated from the intermediate intron by a frame-shift due to an 8-bp deletion of the intermediate intron of *ghsr*. GHS-Rtv is found in chickens (31). The signature “tv” was first used by Sirotkin et al. (31), although its meaning is unclear. The composition of GHS-Rtv is complex: two distinct parts of the intermediate intron sequence of *ghsr* lie between the exon 1 and exon 2 sequences of GHS-R1a [see Ref. (33)]. Kitazawa et al. (32) reported a receptor similar to chicken GHS-Rtv in the Japanese quail. Because the composition was different from that of GHS-Rtv, it was designated as a GHS-Rtv-like receptor and considered to be a possible ortholog of GHS-Rtv. The functions of these avian variants are completely unknown.

Kitazawa et al. (32) reported five isoforms of GHS-Rs in the Japanese quail: GHS-R1a-L, 1a-S, 1aV-L, 1b-L, and 1bV-L. The “L” and “S” appended to GHS-R1a signify the long-type (354 AAs) and short-type (347 AAs) receptors for GHS-R1a, respectively. GHS-R1a-S is a receptor that lacks 7 AAs at the N-terminus of GHS-R1a-L. Two ATG initiation codons are present in the cDNA and the functional codon is unknown.

## Tissue Expression of Ghrelin Receptor mRNAs and Their Isoforms

### Expression of GHS-Ra and GHS-R1a-LR

In agreement with a wide range of physiological functions of ghrelin, GHS-R1a transcripts have been detected in human tissues such as the brain, heart, lung, liver, kidney, pancreas, stomach, intestines, and adipose tissue (34, 35). In particular, high expression levels have been detected in the pituitary gland (36), which is consistent with the role of ghrelin in regulating GH release. In the brain, where expression levels are relatively high, GHS-R1a mRNA is widely distributed in regions linked to energy homeostasis such as the arcuate nuclei of the hypothalamus; area postrema; nucleus of the solitary tract; the dorsal motor nucleus of the vagus; hippocampus; dopaminergic neurons in the ventral tegmental area and substantia nigra; parasympathetic preganglionic neurons; the dorsal and medial raphe nuclei; and the dentate gyrus (9, 34, 37, 38).

In non-mammalian vertebrates, GHS-R1a or GHS-R1a-LR transcripts have been found in the central nervous system and various peripheral organs. As in humans, predominant expression occurs in the pituitary in channel catfish (39), chickens (29, 30, 40–43), and ducks (44) for GHS-R1a, as well as in the black porgy (28), orange-spotted grouper (45), and rainbow trout (23) for GHS-R1a-LR. However, expression in the pituitary gland is not dominant in all species. In Mozambique tilapia, GHS-R1a-LR mRNA is mainly detected in the brain. The distribution of the ghrelin receptor in other tissues also differs among animal species.

In fish, GHS-R transcripts have been detected in most organs. The genes are expressed in all regions of the brain, including the olfactory bulbs and tracts, telencephalon, diencephalon, optic tectum, vagal lobe, hypothalamus, cerebellum and medulla, and spinal cord. Gene expression has also been detected in the eyes, heart, thymus, liver, stomach, intestine, spleen, gill, gall bladder, muscle, kidney, head kidney, Brockmann bodies, skin, muscle, and gonads (23, 26, 28, 39, 45, 46). In rainbow trout, GHS-R1a-LR mRNA expression has been detected in blood leukocytes and head kidney leukocytes (47). Cypriniformes such as goldfish and zebrafish, as well as Siluriformes such as channel catfish, possess paralogs of GHS-Ra, each of which has different levels and patterns of expression (22, 39, 46).

In amphibians, strong GHS-R1a mRNA expression has been found in brain regions such as the diencephalon and mesencephalon; the stomach and testis; and to a lesser extent in the small and large intestines, adrenal gland, and kidney in the bullfrog (19). In the Japanese tree frog, GHS-R1a transcripts have been detected in almost all tissues examined, although relatively high expression was detected in the duodenum, small and large intestines, and ovary. However, unlike in other animals, pituitary expression was absent in both species (19).

In birds, GHS-R1a mRNA has also been detected in almost all tissues examined. GHS-R1a mRNA is expressed in chicken tissues such as the hypothalamus, telencephalon, cerebrum, cerebellum, optic lobes, brainstem, heart, lung, thymus, liver, spleen, pancreas, proventriculus, gizzard, duodenum, adrenal gland, kidney, gonads, breast muscle, subcutaneous fat, leg muscle, abdominal fat, and uropygial gland (29, 30, 40, 41, 44). Comparing the relative expression levels in these tissues is difficult; nonetheless, the expression levels in the brain, gastrointestinal tract, liver, and spleen appear to be relatively high compared with other tissues, although strain differences may exist (29, 30, 33). In ducks, mRNA expression has been detected in the subcutaneous fat, hypothalamus, small intestine, testis, cerebellum, and cerebrum (44). In the Japanese quail, GHS-R1a mRNA expression was examined only in the gastrointestinal tract (32), where region-specific expression was detected at relatively high levels in the upper and lower intestines such as the esophagus, crop, and colon, but weak levels in the middle portions of the gastrointestinal tract (e.g., the proventriculus, duodenum, gizzard, jejunum, and ileum).

### Expression of ghrelin receptor isoforms other than GHS-Ra and GHS-R1a-LR

Growth hormone secretagogue-receptor type-1b is a splice variant of the mammalian GHS-R. In humans, its mRNA distribution is more widespread than that of GHS-R1a, and varies spatially and quantitatively from that of GHS-R1a (34). This suggests the possibility that GHS-R1b is involved in specific GHS-R1a-independent physiological activities, although these remain unknown.

In non-mammalian vertebrates, there are a few reports on the mRNA distribution of GHS-R1b. First, GHS-R1b mRNA has been detected in the brain of fish. In the black porgy, the level of expression was highest in the telencephalon, followed by the hypothalamus, pituitary, optic tectum, thalamus, and spinal cord, whereas little was detected in peripheral tissues (28). In Mozambique tilapia, the brain is the site with the highest expression of GHS-R1b mRNA, although transcripts were also detected in the stomach, adipose tissue, gill, liver, intestine, spleen, kidney, and muscle (26). In orange-spotted grouper, the expression levels of GHS-R1b mRNA were high in the pituitary, hypothalamus, cerebellum, medulla, spinal cord, gill filament, spleen, liver, stomach, head kidney, kidney, gonad, red muscle, skin, and fat body (45). In rainbow trout, GHS-R1b mRNA was strongly expressed in the pituitary, whereas weak expression was observed in the hypothalamus, pyloric appendage, middle intestine, spleen, and head kidney (23). In channel catfish, the expression level of GHS-R1b mRNA was highest in the pituitary, but it was approximately 400 times lower in most peripheral tissues compared with the expression level of GHS-R1a (39).

In birds, GHS-R1aV or GHS-Rtv mRNA expression was detected in almost all tissues examined, a pattern almost identical to that of GHS-R1a mRNA expression, although expression levels of each isoform differed (29, 30, 33). GHS-Rtv transcripts were first detected in chicken ovaries (31). In Japanese quail, the expression of the GHS-Rtv-like receptor was detected in the gastrointestinal tract but only in the proventriculus and gizzard (32). The function of these avian variants is entirely unknown.

## Regulation of Ghrelin Receptor Expression

Satiation and hunger signals regulate *ghsr* expression. A condition of negative energy balance such as fasting increases GHS-R1a mRNA expression in the hypothalamus and pituitary of rats, while re-feeding restores the increased expression level to a normal level (48, 49). The gene expression of *ghsr* is affected by various hormonal factors, it is stimulated by ghrelin (5, 49–51), GH-releasing hormone (GHRH) (52), thyroid hormone (53), and glucocorticoid (dexamethasone) (54, 55). In contrast, it is inhibited by GH (56–58), leptin (49), glucocorticoid (50), and insulin-like growth factor-I (IGF-I) (59). These are summarized in Table 3.

**Table 3:** **Regulation of ghrelin receptor expression**.

Stimulus	Animals (organs)	Receptor, regulation	Reference
Food deprivation	Rats (hypothalamus, pituitary)	1a, ↑	(48, 49)
GHRH	Rats (pituitary)	1a, ↑	(52)
TH	Rats (pituitary)	1a, ↑	(53)
Dexametasone	Rats (hypothalamus, pituitary)	1a, ↑	(54, 55)
L-692,585	Rats (pituitary)	1a, ↓	(52)
GH	Rats (hypothalamus, pituitary)	1a, ↓	(56 –58)
Leptin	Rats (hypothalamus)	1a, ↓	(49)
Adrenalectomy	Rats (hypothalamus, pituitary)	1a, ↓	(54, 55)
Glucocorticoids	Humans (recombinant receptor in GH3 cell)	1a, ↓	(50)
IGF-I	Rats (hypothalamus)	1a, ↓	(59)
Food deprivation	Tilapia (brain)	1a-LR, →	(60)
		1b, ↑	
		Pre-GHS-R, →	
Pre-prandial	Tilapia (brain)	1a-LR, ↑	(60)
Post-prandial		1a-LR, ↓	
Glucose loading	Tilapia (brain)	la-LR, ↓	(62)
14-days starvation	Atlantic salmon (brain)	la-LR, →	(65)
7-days starvation	Goldfish (vagal lobe)	1a-1, ↓ 1a-2, ↓	(22)
10-days starvation	Bullfrog (stomach, ventral skin)	1a, ↑	(19)
10-days dehydration	Japanese tree frog (brain stomach, ventral skin)	1a, ↑	
Catfish GHRL-Gly	Channel catfish (pituitary)	1a, ↑	(39)
		2a, →	
	Channel catfish (Brockman bodies)	2a, ↑	
Catfish GHRL-amide	Channel catfish (pituitary)	1a, →	
		2a, →	
	Channel catfish (Brockman bodies)	2a, ↑	
Goldfish GHRL 12-amide	Zebrafish (brain)	1a, ↑	(46)
		2a, ↑	
Rat ghrelin	Orange-spotted grouper (hypothalamus, pituitary)	1a-LR, ↓	(45)
		1b, ↓	
Chiken ghrelin	Chickens (pituitary)	1a, ↓	(29)
		1aV, ↓	
GHRP-6	Black porgy (recombinant in HEK293)	1a-LR, ↑	(68)
Sea bream GH	Orange-spotted grouper (hypothalamus)	1a-LR, →	(45)
	Orange-spotted grouper (pituitary)	1a-LR, ↓	
	Orange-spotted grouper (hypothalamus, pituitary)	1b, ↓	
Bovine GH	Chickens (pituitary)	1a, ↓	(29)
		1aV, ↓	
Corticosterone	Chickens (pituitary)	1a, ↓	(29)
		1aV, ↓	
Human GHRH 1-29	Chickens (pituitary)	1a, ↓	(29)
		1aV, →	

Acute or chronic changes in the energy status or environmental conditions appear to have varying effects on *ghsr* expression in non-mammalian vertebrates (Table 3). In Mozambique tilapia, GHS-R1a-LR mRNA levels in the brain are unaffected by fasting, whereas GHS-R1b mRNA expression is increased (60). Peddu et al. (61) reported acute pre- and post-prandial changes in GHS-R1a-LR and GHS-R1b mRNA expression, whereas pre-GHS-R mRNA levels (immature mRNA, hetero-nuclear RNA) did not reflect changes in feeding status. Riley et al. (62) showed that acute increased blood glucose reduced GHS-R1a-LR mRNA levels in the brain and increased gastric ghrelin mRNA expression as well as plasma ghrelin levels. This change in plasma ghrelin levels is the opposite of that seen in humans or goldfish, where a glucose load decreases plasma ghrelin levels (63, 64). In conditions of chronic negative energy balance, there was no change in the GHS-R1a-LR expression levels in the brains of Atlantic salmon fasted for 14 days (65). In contrast, goldfish GHS-R1a-1 mRNA levels decreased in the vagal lobe and GHS-R1a-2 mRNA levels increased in the liver after 7 days of fasting (22). In bullfrogs, GHS-R1a mRNA expression was up-regulated in the stomach and ventral skin, whereas that in the brain did not change after 10 days of starvation (19). These results suggest that the nutritional condition of the body affects ghrelin receptor expression. Furthermore, GHS-R1a mRNA expression was up-regulated in the brain, stomach, and ventral skin after 10 days of dehydration of tree frog (19). This result may support the view that ghrelin is involved in the regulation of water balance in frogs, as seen in rats (66) and chicks (67).

Hormonal control of *ghsr* expression has been reported. Ghrelin appears to have a stimulatory effect on *ghsr* expression in non-mammalian vertebrates, as it does in mammals. However, the effects differ depending on the ghrelin form, receptor isoform, and target tissue. In channel catfish, the C-terminal structure of ghrelin affects *ghsr* expression (39). In the pituitary, catfish ghrelin-Gly (this is naturally occurring 23-AA ghrelin where Gly is extended at the C-terminus) increased the levels of GHS-R1a mRNA but not of GHS-R2a mRNA. In contrast, catfish ghrelin-amide (22-AA ghrelin with an amide structure at the C-terminus) had no effect on either receptor. In the Brockmann bodies, catfish ghrelin-amide or ghrelin-Gly dramatically increased the GHS-R2a mRNA expression levels with different time courses. In zebrafish, goldfish ghrelin12-amide stimulated the mRNA expression of both GHS-R1a and 2a in the brain, but with different time courses (46). In orange-spotted grouper, rat ghrelin (10^−5^ M) inhibited the expression of GHS-R1a-LR and GHS-R1b mRNA in the hypothalamus and pituitary (45). In chickens, Geelissen et al. (29) reported that ghrelin down-regulated GHS-R1a and GHS-R1aV mRNA expression in the pituitary *in vitro*. In another *in vitro* study, GHRP-6 stimulated the promoter activity of black porgy GHS-R1a-LR expressed in HEK293 cells (68).

The effects of GH or glucocorticoids on non-mammalian *ghsr* expression also vary depending on the GH species used, target tissue, and GHS-R isoform. In orange-spotted grouper, sea bream GH (10^−7^ M) did not affect GHS-R1a-LR levels in the hypothalamus but reduced them in the pituitary, whereas it decreased GHS-R1b mRNA levels in both the hypothalamus and pituitary (45). In chickens, bovine GH and corticosterone decreased mRNA expression of both GHS-R1a and GHS-R1aV, but human GHRH1-29 reduced only GHS-R1a mRNA expression in the pituitary *in vitro* (29).

Yeung et al. (68) analyzed the 5′-flanking region of *ghsr* in black porgy and identified a number of putative binding sites for transcription factors such as AP1, NF-1, Oct-1, and USF. Changes in *ghsr* expression during embryogenesis have been reported in orange-spotted grouper (45) and channel catfish (39). In both species, *ghsr* expression fluctuates depending on the embryonic stage, and the expression levels of GHS-R isoforms are separately regulated.

## Signaling Pathways of the Ghrelin Receptor

Howard et al. (3) observed increases in intracellular Ca^2+^ levels in cells transfected with GHS-R1a. The intracellular signaling of GHS-R1a is mediated by the activation of a G-protein subtype, Ga_q/11_, which induces the production of inositol triphosphate (IP3), release of Ca^2+^, and activation of protein kinase C (PKC) (69). These events are seen in cells transfected with GHS-R1a as well as in somatotrophs (70–74).

In addition, GHS-R1a functions in an agonist-independent manner and causes high basal IP3 production in the absence of agonists, indicating that GHS-R1a is a constitutively active receptor (71, 74, 75). This activity in turn triggers phospholipase C (PLC)–PKC-dependent Ca^2+^ mobilization, which is associated with the L-type voltage-gated calcium channel via PKC. Furthermore, extracellular signal-regulated kinase 1 and 2 (ERK1/2) are activated by GHRP-6. A GHS-R antagonist (d-Lys3)-GHRP-6, was shown to inhibit basal PLC and ERK1/2 activity (76).

When a non-mammalian ghrelin receptor was expressed in mammalian cells, a rise in intracellular Ca^2+^ was observed with ghrelin or GHSs (19, 22, 27, 28, 32, 77, 78). A similar Ca^2+^ mobilization was also induced by ghrelin in the primary culture of goldfish pituitary cells (79, 80), which was important for inducing the release of GH and luteinizing hormone (LH) from goldfish somatotrophs (79) and gonadotrophs (80), respectively. Little is known about the intracellular signaling pathways involved.

**Figure 5:**
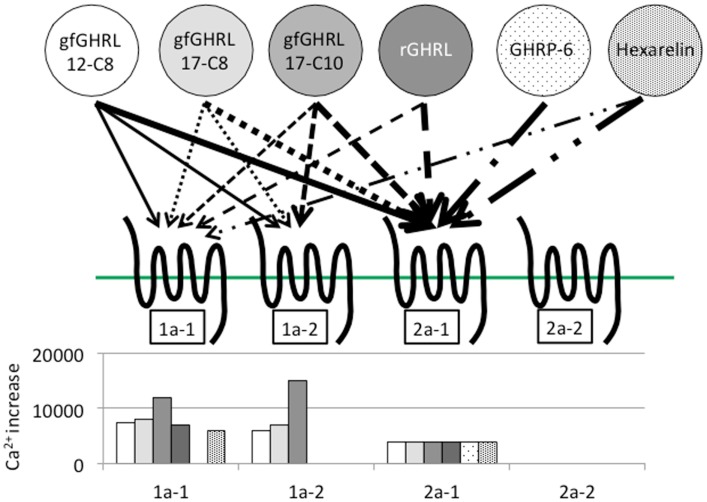
**Ligand selectivity and intracellular Ca^2+^ signaling in four goldfish ghrelin receptors**. Four goldfish ghrelin receptors exhibited different ligand selectivity. The schematic figures above show the strength of the ligand-receptor affinity based on the thickness of the arrow, while the bar graphs below show the maximum value of the stimulated increase in the intracellular Ca^2+^ signal. Goldfish ghrelin (gfGHRL) 12-C8 (octanoylated ghrelin with 12 amino acids, AAs), 17-C8 (octanoylated ghrelin with 17 AAs), and 17-C10 (decanoylated ghrelin with 17 AAs); rat ghrelin (rGHRL); and two GHSs, GHRP-6 and hexarelin, were used in the experiment. For example, the arrows indicate that the intracellular Ca^2+^ increased in cells expressing GHS-R1a-1 after exposure to gfGHRL12-C8, 17-C8, and 17-C10; rat ghrelin; and hexarelin, but not after exposure to GHRP-6 at a similar dose. The corresponding bar graph shows that gfGHRL17-C10 increased Ca^2+^ much more strongly than the other agonists. Furthermore, although GHS-R2a-2 was capable of binding all of the agonists examined at a low dose, none of the agonists increased the intracellular Ca^2+^ level.

In addition to binding ghrelin, non-mammalian ghrelin receptors are capable of binding GHSs such as GHRP-2 and GHRP-6; ipamorelin; and L163,255, L692,585, and L163,540, although the agonistic activity varies according to the receptor present in each animal (19, 22, 27, 28, 32, 77). In addition, a GHS-R1a antagonist (d-Lys3)-GHRP-6, is also capable of inhibiting ghrelin binding to the receptor (22). These results indicate that the structural interactions between the ligand and the AAs of the receptor essential for ligand binding and receptor activation are conserved among vertebrates. However, ligand selectivity has been found in the case of GHRP-6 and hexarelin for goldfish GHS-R1a-1, 1a-2, and 2a-2 (Figure 5) (22).

In fish-specific GHS-R1a-LRs, particularly of the pufferfish and black porgy, pharmacological doses of receptor agonists are required in some cases to activate the receptors (27, 28), whereas no reaction was found at all in the receptors in tilapia and rainbow trout, even with homologous ghrelin (23, 26). The reason behind this phenomenon remains to be elucidated.

Receptor functionality has not been examined in the African clawed frog or teleosts such as channel catfish, zebrafish, and Jian carp where GHS-Ra has been identified. We expect that these receptors will be responsive to ghrelin or GHS because of their structural properties, such as the short ECL2 loop (Figure 4). However, confirmation of these receptor activities will be required to test this hypothesis in the future.

## Key Amino Acids Related to Ligand Selectivity and Receptor Functionality in the Ghrelin Receptor Structure

Feighner et al. (81) reported key AAs that play essential roles in GHS-R1a activation on the basis of the structure of human GHS-R1a and three types of GHSs with different structures, i.e., MK-0677, GHRP-6, and L692,585. Their results showed that D99, C116, E124, M213, S217, and H280 in human GHS-R1a have crucial roles in receptor activation. In particular, M213 is required for the binding of GHRP-6 and L692,585. S217 and H280 are specifically involved with the binding of GHRP-6. In ghrelin receptors identified in non-mammalian vertebrates, all of the AAs listed above are conserved, with the exception of an AA that is equivalent to S217 in the stickleback receptor (Figure 3). This may suggest that the GHS-Ra and GHS-R1a-LR identified in non-mammalian vertebrates have the ability to bind GHSs. However, as described earlier, goldfish GHS-Ra has ligand selectivity (22). In addition, the GHS-R1a-LR in rainbow trout and tilapia shows no Ca^2+^ response in receptor-expressing mammalian cells (23, 26). Although AAs equivalent to M213, S217, and H280, which are essential for binding of GHRP-6 to the receptor, are all conserved in goldfish GHS-Ra, GHRP-6 does not increase the intracellular Ca^2+^ in HEK 293 cells expressing goldfish GHS-R1a-1 and 1a-2. Thus, the interaction between the ligand and key AAs in the receptor related to ligand binding may be more complicated than anticipated.

Holst et al. (82) found that the ghrelin receptor elicited strong, ligand-independent signaling in transfected COS-7 or HEK293 cells. Independent of ligand selectivity, the relationship between constitutive receptor activity and the AA composition of the receptor has also been examined (83–85). These studies suggest that of the AAs in human GHS-R1a, V160, F279, A204, I134, and A204 are important for controlling constitutive receptor activity (Figure 3). These AAs are conserved in the GHS-Ra and GHS-R1a-LR identified in non-mammalian vertebrates (Figure 3); therefore, all of them may be constitutively active receptors, although their activity has been confirmed only in the black porgy receptor (86).

## Physiological Function of GHS-Rs

GHS-R1a mediates the information conveyed by ghrelin and elicits various physiological functions. In addition to its hypophysiotropic effects and regulation of appetite, ghrelin affects many physiological functions, including gastrointestinal motility, cardiovascular performance, cell proliferation, immune function, bone metabolism, sleep, and the promotion of learning and memory (9, 87, 88). Recent evidence suggests that ghrelin functions as a blood glucose regulator (89).

### Roles of GHS-R1a and 2a

Growth hormone secretagogue-receptor type-1a or 2a is thought to mediate various physiological functions of ghrelin, although direct evidence in non-mammalian vertebrates remains sparse. Recently, Yahashi et al. (90) reported that the peripheral effects of ghrelin on food intake and locomotor activity in goldfish are mediated via one of the four ghrelin receptor isoforms, GHS-R2a-1. In addition, ghrelin has the ability to stimulate GH and LH release from goldfish pituitary (64, 79, 80, 91). GHS-R1a-2 mRNA shows the most abundant expression in this structure, suggesting that the receptor is involved in the regulation of pituitary hormone release. Changes in GHS-R1a or 2a expression depending on the energy state suggest the involvement of ghrelin in energy homeostasis, as observed in frogs and goldfish (19, 22). However, no change was observed in the case of tilapia (60). In chickens and quails, the distributions of the receptor are consistent with its role in gut contraction (32). However, although the ghrelin receptor is expressed throughout the intestinal tracts of goldfish and rainbow trout, ghrelin has no effects on intestinal motility (92). This result is in contrast to that seen in zebrafish, in which rat and human ghrelin stimulate gut contraction (93). Further studies are necessary to determine the nature of the relationship between ghrelin receptors and physiological function.

### Roles of GHS-R1b

In contrast with GHS-R1a, little is known about the functions of the GHS-R1b isoform. Mammalian and non-mammalian GHS-R1b show no apparent intracellular Ca^2+^ signaling response to ghrelin or GHSs (32, 86). Co-expression of GHS-R1a and 1b reduces the signaling capacity of GHS-R1a via hetero-dimerization (28, 86, 94), suggesting that GHS-R1b acts as a dominant-negative mutant during signaling via GHS-R1a (86). Intriguingly, GHS-R1b forms heterodimeric associations with other GPCRs such as neurotensin receptor 1 (NTSR1) (95). This heterodimeric receptor binds to peptide hormones other than ghrelin and affects intracellular signaling, i.e., the GHS-R1b/NTSR1 heterodimer binds neuromedin-U and induces cAMP production instead of Ca^2+^ signaling.

Although GHS-R1b exists in the same gene as GHS-R1a, the sites, patterns, levels, and regulation of GHS-R1b expression differ from those of GHS-R1a. Therefore, elucidation of the physiological function of the receptor is awaited.

## Perspective

In this review, we assembled current knowledge about ghrelin receptors in non-mammalian vertebrates. Many questions remain unanswered because receptor genes have been identified only in a limited number of species. However, the functional importance of the ghrelin system is gradually becoming understood in species where the receptor distribution is clear. Presence of unique GHS-Rs such as GHS-R2a, GHS-R1a-LR, or variants found only in non-mammalian vertebrates are interesting in the divergence of the ghrelin system; therefore, examining the structural relationship and function of non-mammalian GHS-Rs based on comparisons with mammalian GHS-Rs is important for understanding the significance of the ghrelin system in vertebrates. However, the ghrelin system of an animal studied may also need to be considered without preconceptions or making comparisons with mammalian data. Thus, the study of non-mammalian GHS-Rs should be interesting and attract many researchers in the future.

## Conflict of Interest Statement

The authors declare that the research was conducted in the absence of any commercial or financial relationships that could be construed as a potential conflict of interest.
